# Hoxa5 alleviates obesity‐induced chronic inflammation by reducing ER stress and promoting M2 macrophage polarization in mouse adipose tissue

**DOI:** 10.1111/jcmm.14600

**Published:** 2019-08-23

**Authors:** Weina Cao, Tiantian Zhang, Ruonan Feng, Tianyu Xia, Hongtao Huang, Chenlong Liu, Chao Sun

**Affiliations:** ^1^ College of Animal Science and Technology Northwest A&F University Yangling Shaanxi China; ^2^ Faculty of Animal Science and Technology Yunnan Agricultural University Kunming Yunnan China

**Keywords:** adipocyte, endoplasmic reticulum stress, Hoxa5, macrophage, obesity

## Abstract

Obesity‐induced chronic inflammation is associated with endoplasmic reticulum stress (ERS) in adipocytes and changes in both the number and phenotype of adipose tissue macrophages (ATMs). In addition, ERS enhances macrophage activation. So far, the function of Hoxa5 in obesity‐induced chronic inflammation has been poorly understood. Herein, we demonstrate the importance of the transcription factor, Hoxa5, in determining adipose tissue macrophage (ATM) polarity and ERS. Hoxa5 decreased bodyweight, reduced inflammatory cytokine secretion and corresponded with an increased number of M2 macrophages in the adipose tissue of high‐fat diet (HFD) mice. Transcriptome sequencing data showed that overexpression of Hoxa5 in adipocytes changed expression of endoplasmic reticulum (ER) protein processing‐related genes. Based on transcriptome sequencing data and bioinformatics prediction, we have been suggested that Hoxa5 alleviated inflammatory responses by inhibiting ERS and by activating PPARγ pathway in mouse adipose tissue. Hoxa5 alleviated ERS and inflammatory responses by inhibiting the eIF2α/PERK signalling pathway in adipocytes. Hoxa5 also inhibited chronic inflammation of adipocytes by promoting M2 macrophage polarization. In addition, Hoxa5 transcriptionally activated the PPARγ pathway to promote polarization of M2 macrophages, which in turn alleviated chronic inflammation of adipocytes. Taken together, these results shed light on the mechanisms underlying Hoxa5‐dependent inhibition of obesity‐induced chronic inflammation by reducing ERS and promoting polarization of M2 macrophages. These results suggest that Hoxa5 may be a potential therapeutic target for obesity and other metabolic syndromes.

## 1 INTRODUCTION

Obesity has become a universal health problem worldwide and is considered to be a chronic inflammatory state that is characterized by the infiltration and activation of immune cells in metabolic organs, such as adipose tissue.^1^, ^2^, ^3^ It is well known that metaflammation induced by over‐nutrition of adipose tissue has been recognized as an important link in the pathogenesis of insulin resistance and type 2 diabetes.^4^

Various congenital and adaptive immune cells interact with adipocytes to maintain adipose tissue.^5^ In particular, adipose tissue macrophages (ATMs) are primary effectors of inflammation and are essential for the co‐ordination of metabolic inflammation. ATMs exist in a range of functionally different activation states and respond to multiple metabolic signals, exerting profound regulatory functions on metabolism.^6^, ^7^, ^8^, ^9^, ^10^ It has been reported that ATMs are present in crown structures (CLS) around dead fat cells in obese individuals and exhibit a major proinflammatory phenotype consistent with classical activation of Ml macrophages.^1^ Furthermore, polarization of M1 macrophages provokes insulin resistance and type 2 diabetes.^8^, ^9^, ^11^ In contrast, ATMs are evenly dispersed in lean mice and primarily display an anti‐inflammatory selective activation (M2) phenotype^12^; polarization of M2 macrophages enhances adaptive thermogenesis and energy expenditure.^13^, ^14^, ^15^ It is worth noting that activation of the PPARγ signal pathway in macrophages promotes M2 macrophage polarization.^16^, ^17^

The ER is an important organelle for cell nutrition induction and transmission.^2^ ERS is thought to play a key role in the development of obesity‐associated insulin resistance.^18^ Excessive protein folding events trigger ERS and activate the unfolded protein response (UPR). The three UPR pathways co‐ordinately restore ER homeostasis and affect numerous aspects of cell functions.^19^, ^20^, ^21^ Notably, the transcription factor, C/EBP homologous protein (CHOP), is a downstream component of the ERS pathway. It has been reported that consuming a high‐fat diet results in a similar number of infiltrating macrophages into white adipose tissue (WAT). In CHOP−/− mice, the infiltrating macrophages are primarily M2 variants and result in remission of insulin resistance.^22^

The Homeobox a5 (Hoxa5) gene is an important developmental transcription factor that is highly expressed in adipose tissue.^23^ Previous studies reported that lung inflammation was observed in Hox5 mutant mice, suggesting that Hoxa5 is involved in vascular inflammation.^24^, ^25^ In addition, we previously confirmed that Hoxa5 promotes white adipose tissue browning by inhibiting lipopolysaccharide (LPS)‐induced inflammation in mice.^26^ However, the regulatory effect of Hoxa5 on obesity‐induced chronic inflammation remains unclear.

In the present study, we found that Hoxa5 alleviates ERS by inhibiting the eIF2α/PERK signalling pathway and transcriptionally activated the PPARγ pathway to promote polarization of M2 macrophages. M2 macrophages activation subsequently alleviated obesity‐induced chronic inflammation. Therefore, we explored the intrinsic link between Hoxa5, ERS, chronic inflammation and M2 macrophage polarization to provide a theoretical basis for the treatment of obesity.

## 2 MATERIALS AND METHODS

### 2.1 Animal experiments

Four‐week‐old C57BL/6 male mice were used. The mice were randomly assigned into two groups that were fed either a high‐fat diet or a chow diet for 8 weeks. All mouse handling methods and experimental protocols were followed as previously described.^27^ Hoxa5 adenoviral vector (pAd‐Hoxa5), the purified product of a Hoxa5 adenovirus interfering vector (sh‐Hoxa5) or an empty adenoviral vector (pAd‐Control) was subcutaneously injected at a titre of 1 x 10^12^ IFU/mL into the mice once a day for 10 days. The mice were then killed, and adipose tissue and blood were collected.

### 2.2 Drug treatments

Free fatty acids (50 mg/mL; Sigma‐Aldrich) containing myristic acid, lauric acid, arachidonic acid, oleic acid and linoleic acid were used to treat adipocytes for 4 hours to induce chronic inflammation of adipocytes. The adipocytes were treated with 2 µg/mL tunicamycin (TM; Sigma‐Aldrich) or 1 μmol/L thapsigargin (TG; Sigma‐Aldrich) for 12 hours to induce endoplasmic reticulum stress (ERS). The adipocytes were also incubated with 2 mmol/L tauroursodeoxycholate (TUDCA; MedChemExpress) for 1 hour to induce ERS. Adipocytes were incubated with 50 mmol/L 4‐PBA (Sigma‐Aldrich) for 1 hour to suppress ERS.^28^ The PERK signalling pathway was blocked by 1 μmol/L GSK2656157 (Adooq Bioscience) for 2 hours; IRE1 was blocked by 50 μmol/L STF‐083010 MedChemExpress) for 2 hours. Macrophages were treated with 100 nmol/L rosiglitazone (MedChemExpress) for 24 hours to activate the PPARγ signalling pathway.

### 2.3 Primary adipocyte culture

The preparation of primary pre‐adipocyte culture was as described before.^29^ Briefly, epididymal white adipose tissues from 4‐week‐old mice were harvested, visible fibres and blood vessels were removed, and the adipose tissue was washed three times with PBS buffer containing 200 U/mL penicillin (Sigma) and 200 U/mL streptomycin (Sigma). Then, pre‐adipocytes were seeded onto culture dishes at 30% confluency and incubated at 37°C under a humidified atmosphere of 5% CO_2_ and 95% air until confluence.

Differentiation of pre‐adipocytes was performed as follows. Cells grown to 100% confluence (day 0) were induced to differentiate using DMEM/F12 medium containing dexamethasone (1 mol/L; Sigma), insulin (10 g/mL; Sigma), IBMX (0.5 mmol/L; Sigma) and 10% FBS. Four days after the induction (from day 2), cells were maintained in the induction medium containing insulin (10 g/mL; Sigma) and 10% FBS.

### 2.4 Cell infection and transfection

The pre‐adipocytes were infected with overexpression Hoxa5 adenovirus vector (pc‐Hoxa5) or adenovirus interference vector of Hoxa5 (sh‐Hoxa5) for 48 hours at 1 × 10^9^ IFU/mL. Interference vector of ATF6 (si‐ATF6) or PPARγ (si‐PPARγ) was stored in our laboratory. The X‐tremeGENE HP Reagent (Roche) was used for plasmid transfection. The transfection procedure was according to the manufacturer's instructions.

### 2.5 RNA‐sequence analysis

Adipocytes were harvested from inguinal white adipose tissues of four‐week‐old mice. The adipocytes were inducted for 4 days, differentiated and then infected with purified products of overexpression Hoxa5 adenovirus vector (pc‐Hoxa5) or empty adenovirus vector (control). Total RNA was isolated from adipocytes and used for the RNA‐sequence analysis as previously described.^30^

### 2.6 Correlation analysis

GraphPad Prism 5.0 was used for the correlation analysis between the mRNA expression of Hoxa5 and CHOP. Two‐tailed Pearson's test was chosen, and the confidence interval was 95%.

### 2.7 Flow cytometry

Mouse epididymis WAT was isolated and digested with 0.1% collagenase type I (Sigma‐Aldrich) at 37°C with shaking at 200 rpm for 60–90 minutes. Digested tissues were filtered through a 70‐µmol/L nylon mesh and centrifuged at 500 g for 5 minutes. Floating adipocytes and SVF pellets were collected, and the SVFs were resuspended in red‐blood‐cell lysis buffer (eBioscience RBC Lysis Buffer) before further analysis. To isolate ATMs (CD45+F4/80+) or adipocytes (CD90+), SVFs were washed with PBS and incubated with the desired combination of fluorochrome‐conjugated antibodies, including Brilliant Violet 421 anti‐mouse F4/80 BM8 (BioLegend), FITC anti‐mouse‐CD45 (BioLegend) and PE‐Cy7‐anti‐CD90 (eBioscience) for 30 minutes at 4°C in the dark. To further sort M1 (F4/80+CD11c+CD206−) and M2 (F4/80+CD11c−CD206+) macrophages, APC‐anti‐CD11c (eBioscience) and PE‐anti‐CD206 (AbD Serotec) were used. Validation information on the antibodies is available on the manufacturer's websites. Cells were then subjected to flow cytometric analysis with a BD FACS Aria II flow cytometer (BD Biosciences). Data were analysed with FlowJo version 7.6.4.

### 2.8 Gating strategy to identify and characterize ATMs

Firstly, we used an unstained control sample, adjust side scatter and forward scatter so that the cell populations of interest are on scale. In addition, the unstained control sample was used to adjust the photomultiplier tube (PMT) gain for each fluorochrome detector so that the peak MFI of the unstained cells on a histogram is within 10^1^‐10^2^ on a log scale. Then, we acquired all SS compensation controls. We acquired and save 10 000‐30 000 events for each SS control. At last, we calculated compensation values across all included detector according to instructions for the instrument with applying compensation values to all SS controls, FMO controls and samples of interest.

### 2.9 Immunohistochemistry and immunofluorescence

Frozen sections of inguinal adipose tissue were prepared, and antigen retrieval and endogenous peroxidase activity blocking were performed. Tissues were then incubated with anti‐PGC1α (Abcam), and biotinylated secondary antibodies (1:3000) were used for incubation.

The 10‐µm‐thick frozen sections of adipose tissue or the cells were incubated in 4% paraformaldehyde for 20 minutes and then permeabilized with 0.1% Triton X‐100 for 10 minutes. Non‐specific binding was blocked with 5% BSA for 30 minutes. Frozen sections or cells were incubated with primary antibodies against CHOP (ab11419), CD68 (ab125212), CD163 (ab182422), CD206 (ab64693), TNF‐α (ab8348), IL4 (ab11524), IL10 (ab9969), α‐actin (ab5694) and IFNγ (ab9657) at 37℃ for 2 hours; the primary antibodies were from Abcam. Frozen sections or cells were stained at with a 1:100 dilution of FITC‐conjugated goat anti‐rabbit IgG (Sangon Biotech) in 2% BSA for 30 minutes. All of the washes were performed with 1 × PBS. An anti‐fade solution containing DAPI (Solarbio) was used to dye cell nucleus.

### 2.10 Culture and co‐culture of macrophages

RAW264.7 macrophages were treated with basal medium containing 500 ng/mL LPS (Sigma) for 24 hours to obtain M1 macrophages. M2 macrophages were obtained by treatment with basal medium containing 10 ng/mL IL4 for 24 hours. Polarized M1 or M2 macrophages were washed three times in PBS to remove the LPS. The cells were then cultured in fresh RPMI basal medium for 24 hours. The resulting M1 or M2 macrophage conditioned medium was collected in a sterilized centrifuge tube, centrifuged at 2000 g for 10 minutes to remove cell debris, and the supernatant was saved. This medium was used to grow mature adipocytes for 24 hours, and these co‐cultured, mature adipocytes were harvested for cytokine detection.^31^

### 2.11 Luciferase report assay

Fragments containing PPARγ promoter sequences were subcloned into a pGL3‐basic vector (Takara). HEK293T or 3T3‐L1 cells were cultured in 24‐well plates and cotransfected with either the PPARγ promoter plasmid and pc‐Hoxa5 or pcDNA3.1. After 48 hours, cells were harvested, and luciferase expression was analysed using the Dual‐Luciferase Reporter Assay System following the manufacturer's instructions (Promega).

### 2.12 Electrophoretic mobility shift assay (EMSA)

We prepared nuclear protein extracted from adipocytes as previously described.^32^ EMSA was performed followed according to the instructions in the LightShift Chemiluminescent EMSA Kit (Pierce Corp).

### 2.13 Enzyme‐linked immunosorbent assay (ELISA)

TNF‐α and IL‐6 levels in mouse serum were measured using commercial ELISA kits according to the manufacturer's instructions (Sigma‐Aldrich).

### 2.14 Glucose Tolerance Tests (GTT)

Glucose tolerance tests (GTTs) were performed on staving mice, which were fasting for 10 hours. Glucose (1 g/kg of bodyweight) was administered into the intraperitoneal space of the mice, and blood glucose was assayed immediately before and at 15, 30, 60 and 120 minutes after administration.^33^

### 2.15 Real‐time quantitative PCR analysis

Primers for Hoxa5, CHOP, GRP78, TNF‐α, IL6, PERK, PPARγ, CD206, IL10, XBP1 and ATF6 were synthesized by Invitrogen. Gene‐specific primers used in the qPCR experiments are listed in Supplementary Table, and β‐actin was used as the endogenous control gene. The expression of genes was analysed by the method of 2^−ΔΔCt^. Quantitative PCR was performed as previously described.^34^

### 2.16 Western blot analysis

Antibodies against CHOP (ab11419), GRP78 (ab108615), IRE1 (ab37152), XBP1 (ab37152), ATF6 (ab203119), STAT6 (ab32520), CD163 (ab182422), CD206 (ab8918), IL4 (ab11524), IL10 (ab9969), IL6 (ab7737), TNF‐α (ab8348), Hoxa5 (ab140636) and GAPDH (ab9484) were purchased from Abcam. PERK (3192), eIF2α (5324) and p‐eIF2α (3398) were purchased from Cell Signaling Technology. PPARγ (Ap0686) was purchased from Bioworld. We used GAPDH as internal control gene, and the experimental procedure was described in our previous reports in detail.^35^

### 2.17 Statistical analysis

All statistical analyses were performed using SAS v 8.0 (SAS Institute). Statistically significant differences in the data were assessed using an unpaired t test and one‐way ANOVA. Comparisons among individual means were performed using Fisher's least significant difference (LSD). Data are presented as the mean ± SD values; *P* < .05 was considered to be significant.

## 3 RESULTS

### 3.1 Hoxa5 expression was decreased under high‐fat diet–induced ERS and chronic inflammation in mouse adipose tissue

**Figure 1:**
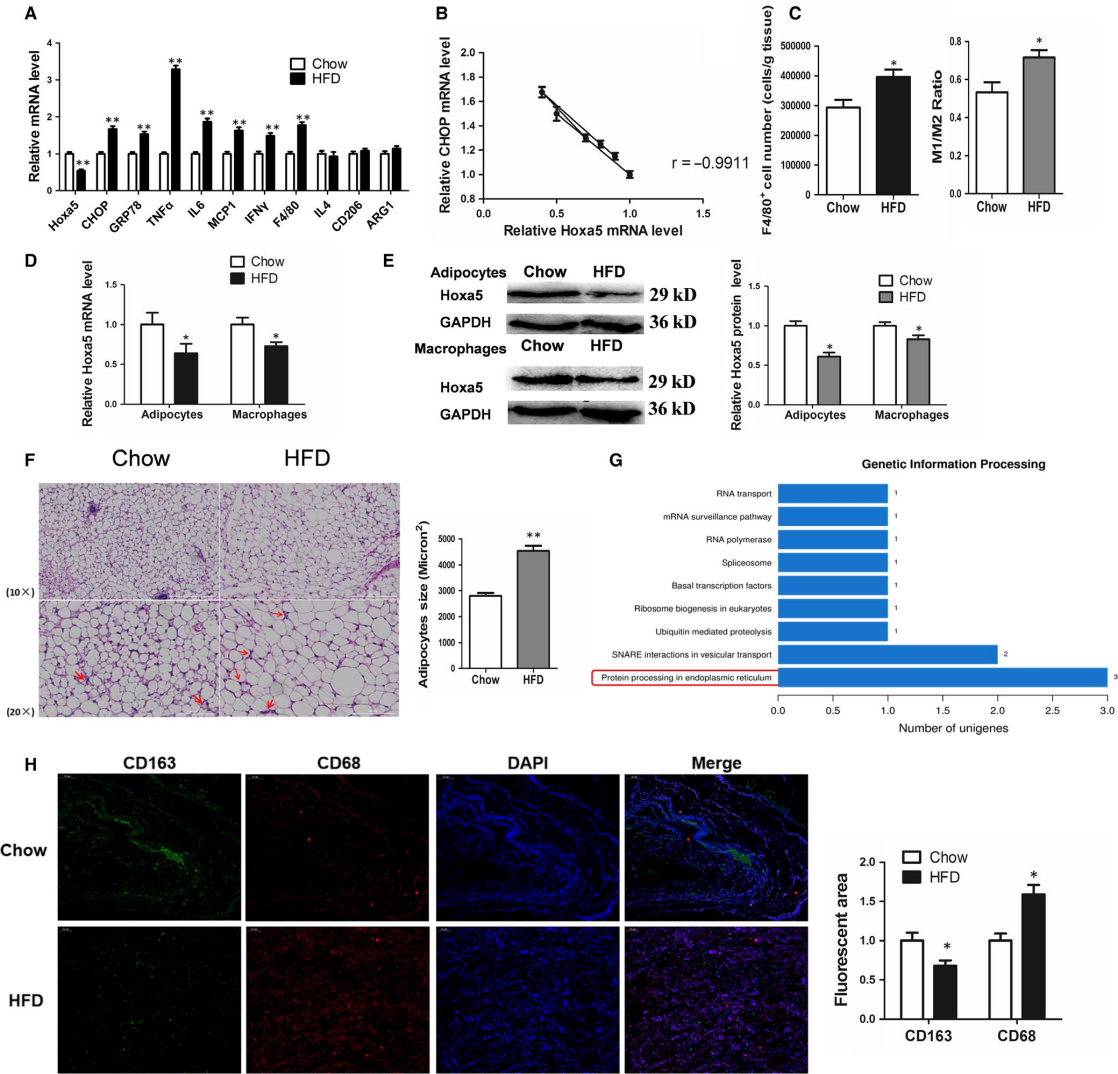
Hoxa5 expression is decreased during high‐fat diet–induced ER stress and chronic inflammation in mouse adipose tissue. (A) Relative mRNA levels of Hoxa5, CHOP, GRP78, TNF‐α, IL6, MCP1, IFNγ, F4/80, IL4, CD206 and ARG1 of inguinal white adipose tissue (iWAT; n = 6). (B) Correlation analysis of CHOP and Hoxa5 mRNA levels in mouse inguinal adipose tissue (iWAT) (n = 18). (C) The ratio of M1/M2 macrophages and the total number of F4/80^+^ cells that were isolated from epididymal adipose tissue (n = 5). (D) Relative mRNA levels of Hoxa5 in adipocytes and macrophages, respectively (n = 5). (E) Relative protein levels of Hoxa5 in adipocytes and macrophages (n = 5). (F) Macrophage number and adipocyte size were observed in frozen sections of iWAT samples by haematoxylin and eosin staining (n = 6). Red arrows show crown structures (CLS) of macrophages. (G) Analysis of genetic information processing of RNA‐sequence in response to overexpression of Hoxa5 in mouse adipocytes (n = 3). (H) Images of iWAT from different groups that were immunostained for CD163 and CD68 (n = 6). The plotting scale is shown in the top left corner of each image. Values are the means ± SD values. *P < .05, **P < .01, compared with the control group

We firstly established an obese mouse model using a high‐fat diet (Figure S1A). Compared with the control group, the serum levels of TNF‐α and triglycerides of obese mice were significantly increased (Figure S1B, S1C; *P* < .01). The GTT assay showed increased insulin resistance in the obese mice (Figure S1D; *P* < .05). The expressions of ER‐associated factors, CHOP and GRP78, and the inflammatory cytokines, TNF‐α, IL6, MCP1 and IFNγ, were significantly up‐regulated, while the expression level of Hoxa5 was significantly down‐regulated in the adipose tissue of obese mice (Figure 1A; *P* < .01). In addition, the general macrophage marker, F4/80, was increased, whereas the M2‐specific macrophage markers, IL4, CD206 and ARG1, were not significantly changed, suggesting that M1 macrophages infiltrated the adipose tissue in the obese state. Interestingly, there was a significant negative correlation between Hoxa5 and expression of the ERS marker, CHOP, in adipose tissue (Figure 1B; *r* = −.9911). We further confirmed the number of macrophages in mouse adipose tissue by flow cytometry. The results showed that the ratio of M1 (F4/80+CD11c+CD206−)/ M2 (F4/80+CD11c−CD206+) macrophages was significantly higher in the HFD mice, and the total macrophages (F4/80+) were also increased in the HFD mice (Figure 1C; *P* < .05). The results showed more macrophages and more M1 macrophages in obese mouse adipose tissue than that of control group (Figure 1G; *P* < .05). The expression pattern of Hoxa5 in flow‐sorted adipocytes or macrophages was determined. In adipocytes and macrophages from the HFD group, Hoxa5 mRNA and protein expression were decreased (Figure 1D,1; *P* < .05). H&E staining of adipose tissue revealed infiltration of ATMs and enlargement of the adipocytes in the obese mice (Figure 1F; *P* < .01). Moreover, we used RNA‐sequence to compare adipocyte transcriptomes that were infected with an adenovirus overexpressing Hoxa5 or a control adenovirus. Overexpression of Hoxa5 changed the expression patterns of ER protein processing‐related genes in adipocytes (Figure 1G). Immunofluorescence staining showed a decrease in CD163‐positive cells and an increase in CD163‐positive cells, indicating the polarization of M1 macrophages in the adipose tissue of HFD mice. (Figure 1H; *P* < .05).

### 3.2 Hoxa5 inhibited inflammatory cytokines secretion by alleviating ERS in mouse adipocytes

**Figure 2:**
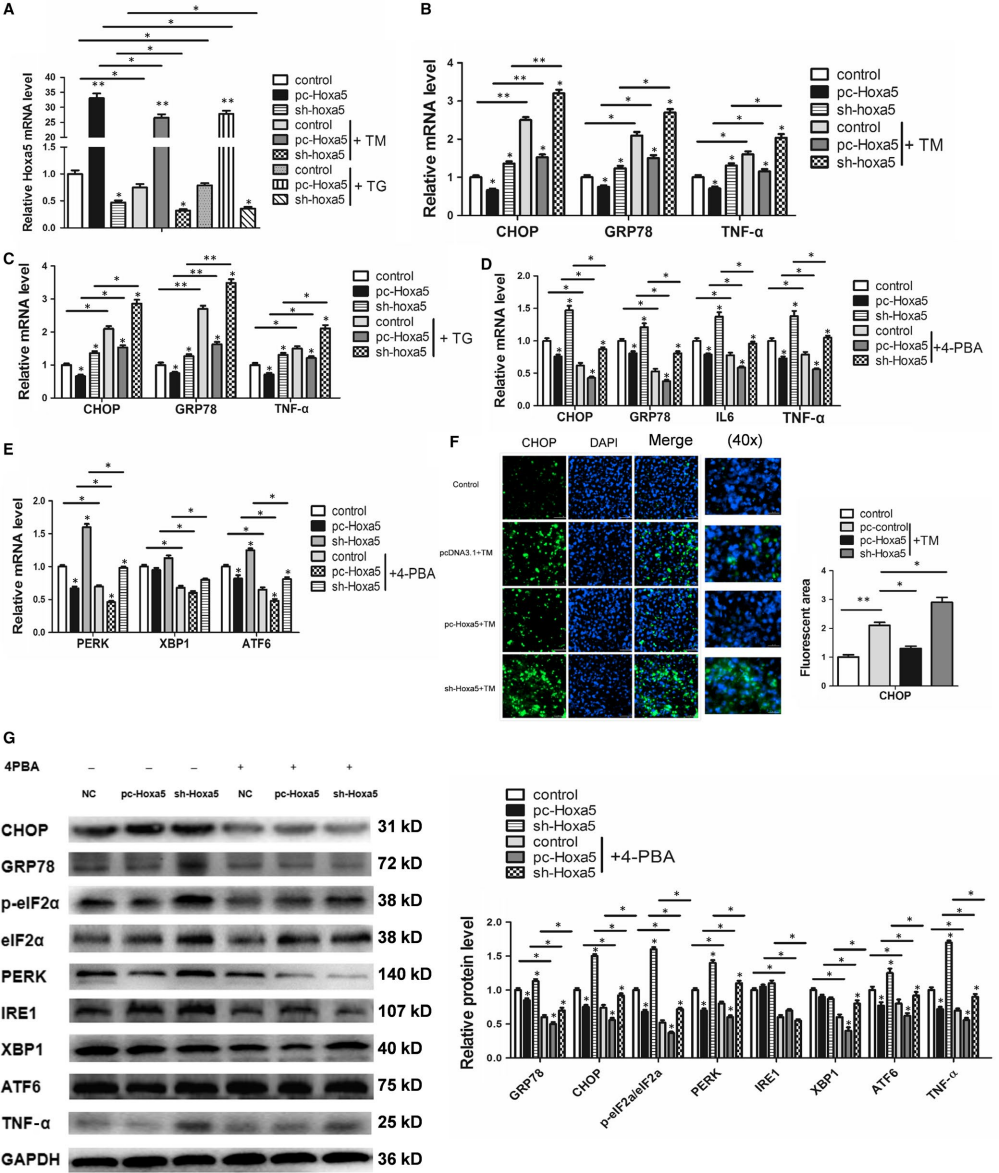
Hoxa5 inhibits inflammatory cytokine secretion by alleviating ER stress in mouse adipocytes. (A‐E,G) Adipocytes were infected with pc‐Hoxa5, sh‐Hoxa5 or pc‐control. (A) Relative mRNA level of Hoxa5 in adipocytes that were incubated with or without tunicamycin (TM) or thapsigargin (TG) (n = 4). (B) mRNA levels of CHOP, GRP78 and TNF‐α in adipocytes that were incubated with or without TM (n = 4). (C) mRNA levels of CHOP, GRP78 and TNF‐α in adipocytes that were incubated with or without TG (n = 4). (D‐G) Adipocytes were infected with pc‐Hoxa5 or sh‐Hoxa5 and incubated with TM. (D,E) mRNA levels of CHOP, GRP78, IL6, TNF‐α, PERK, XBP1 and ATF6 in adipocytes that were incubated with or without 4‐PBA (n = 4). (F) Images showing immunostaining of CHOP in adipocytes incubated with or without TM (n = 4). (G) Relative protein levels of GRP78, CHOP, eIF2α, PERK, IRE1, XBP1, ATF6 and TNF‐α in adipocytes that were incubated with or without 4‐PBA (n = 4). Values are the means ± SD values. *P < .05, **P < .01, compared with the control group

Having found Hoxa5 may be associated with ERS, we then treated the adipocytes with tunicamycin (TM) and thapsigargin (TG) to induce ERS and found that both compounds significantly down‐regulated Hoxa5 expression (Figure 2A; *P* < .05). In both the tunicamycin and thapsigargin‐induced ERS models, Hoxa5 significantly inhibited the expressions of CHOP, GRP78, TNF‐α and IL6 (Figure 2B, 2C; *P* < .05). In the presence of ERS inhibitors (4‐PBA or TUDCA), CHOP, GRP78, TNF‐α and IL6 were decreased by Hoxa5 expression (Figure 2D, Figure S2B; *P* < .05). Interestingly, the effects of Hoxa5 on the expression of key genes involved in the three pathways of ERS were examined under the combined treatment of TM and 4‐PBA. It was found that Hoxa5 inhibited the mRNA expression of PERK and ATF6 (*P* < .05), but has no significant effect on XBP1 (Figure 2E). We further measured the expression of relevant protein factor levels and confirmed that Hoxa5 inhibited the expression of GRP78, CHOP and TNF‐α, and blocked the PERK/eIF2α pathway (Figure 2G; *P* < .05). Immunofluorescence detection showed that overexpression of Hoxa5 inhibited the expression of CHOP (Figure 2F; *P* < .05). We further investigated the effect of Hoxa5 on chronic inflammation in adipocytes. Primary murine adipocytes were treated with free fatty acids to imitate an obesity model in vitro. The result showed that free fatty acids (FFA) significantly inhibited Hoxa5 expression and significantly up‐regulated the expressions of ERS markers, CHOP and GRP78, and the inflammatory cytokines, TNF‐α and IL6 (Figure S2A; *P* < .05). Furthermore, regardless of whether free fatty acids were present, overexpression of Hoxa5 significantly inhibited the expressions of CHOP, GRP78, TNF‐α and IL6 (Figure S2C; *P* < .05). By combined treatment of FFA and 4‐PBA, we found 4‐PBA inhibits the expression of CHOP, GRP78, IL6 and TNF‐α in the process of chronic inflammation induced by FFA (Figure S2D; *P* < .05).

### 3.3 Hoxa5 decreased ERS via blocking PERK/eIF2α signal pathway in mouse adipocytes

**Figure 3:**
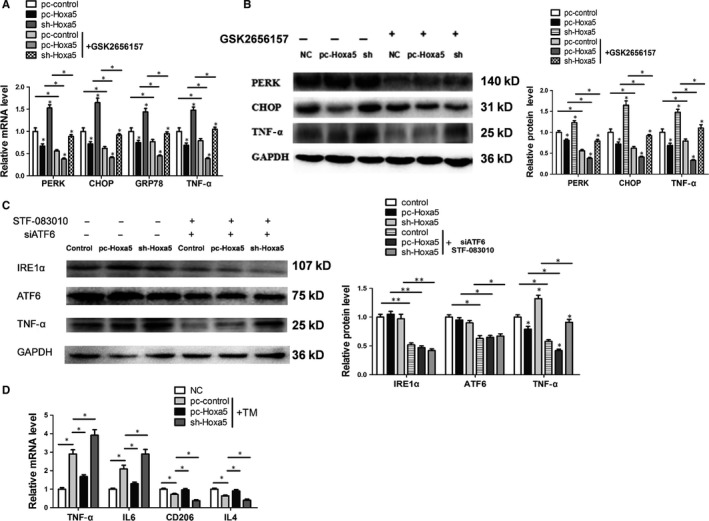
Hoxa5 decreases ER stress via blocking the PERK/eIF2α signalling pathway in mouse adipocytes. (A‐C) Adipocytes were infected with pc‐Hoxa5 or sh‐Hoxa5 and incubated with TM. (A) Relative mRNA levels of PERK, CHOP, GRP78 and TNF‐α in adipocytes incubated with or without GSK2656157 (n = 4). (B) Relative protein levels of PERK, CHOP and TNF‐α in adipocytes incubated with or without GSK2656157 (n = 4). (C) Relative protein levels of IRE1α, ATF6 and TNF‐α in adipocytes treated with or without STF‐083010 and siATF6 (n = 4). (D) Relative mRNA levels of TNF‐α, IL6, CD206 and IL4 in macrophages co‐cultured with adipocytes from different groups incubated with or without TM (n = 4). Values are the means ± SD values. *P < .05, **P < .01, compared with the control group

Having observed inhibitory effect of Hoxa5 on PERK/eIF2α signalling, we further investigated the effects of Hoxa5 on the PERK/eIF2α pathway in adipocytes co‐treated with TM and other related pathway inhibitors. It was found that Hoxa5 significantly reduced both mRNA and protein levels of PERK, CHOP, GRP78 and TNF‐α expression, regardless of the presence of PERK inhibitor treatment (Figure 3A; *P* < .05). Consistently, overexpression of Hoxa5 significantly decreased the protein levels of PERK, CHOP and TNF‐α (Figure 3B). However, upon co‐treatment with the IRE1α inhibitor, STF‐083010 and si‐ATF6, Hoxa5 had no significant effect on the protein expressions of ATF6 and IRE1α. Moreover, the IRE1α inhibitor and si‐ATF6 down‐regulated TNF‐α expression (Figure 3C; *P* < .05). Co‐culturing of adipocytes and macrophages under ERS revealed that the macrophages in the pc‐Hoxa5 group exhibited increased mRNA expression of CD206 and IL4, and reduced levels of TNF‐α and IL6 mRNA (Figure 3D; *P* < .05).

### 3.4 Hoxa5 alleviated chronic inflammation of adipose tissue by promoting the polarization of M2 macrophages

**Figure 4:**
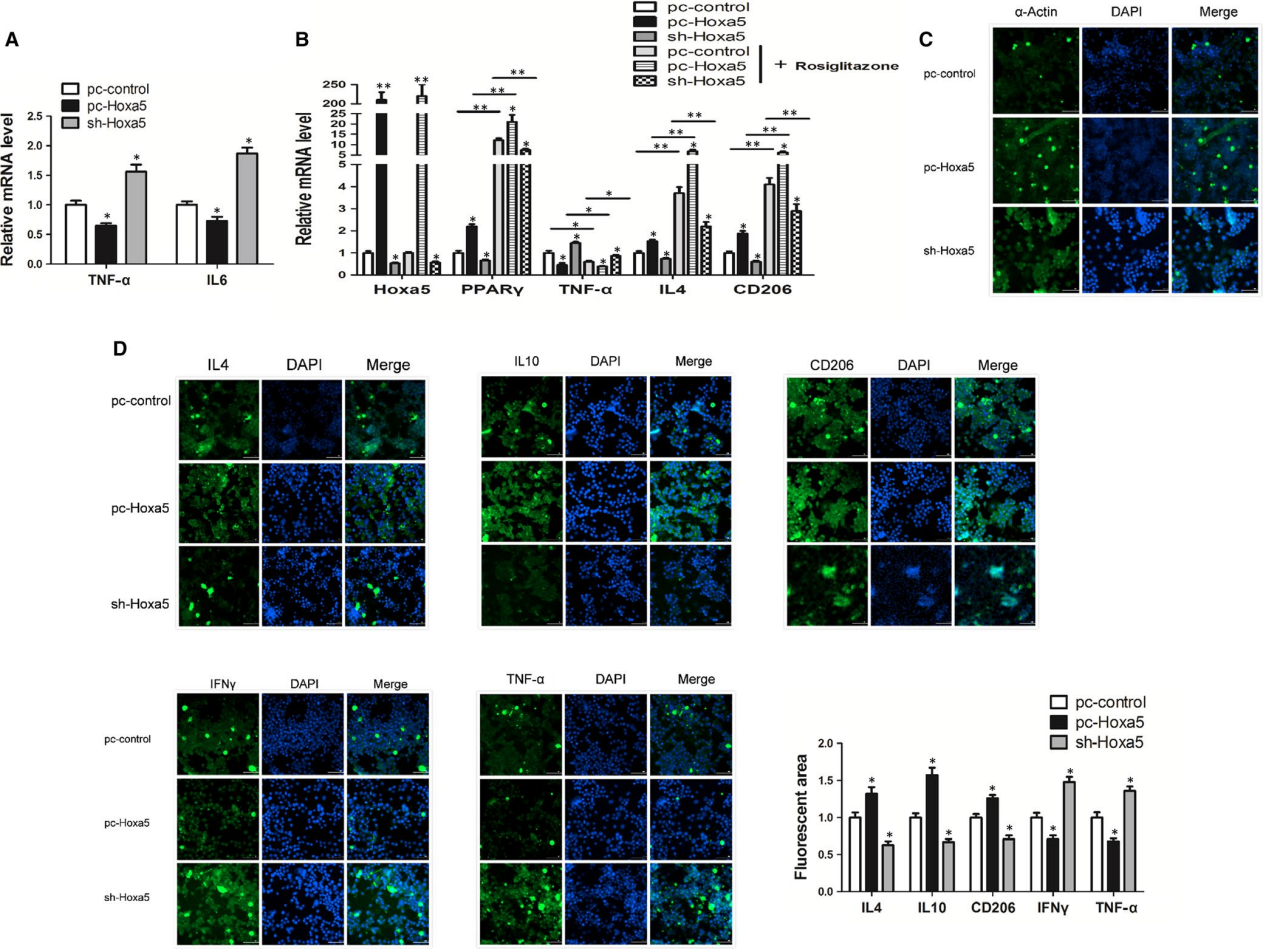
Hoxa5 alleviates chronic inflammation of adipose tissue by promoting M1 to M2 macrophage polarization. (A) Relative mRNA levels of TNF‐α and IL6 in adipocytes co‐cultured with macrophages (RAW264.7) that were transfected with pc‐Hoxa5 or sh‐Hoxa5 (n = 4). (B‐C) Macrophages were transfected with pc‐Hoxa5 or sh‐Hoxa5. (B) Relative mRNA levels of Hoxa5, PPARγ, TNF‐α, IL4 and CD206 in macrophages incubated with or without rosiglitazone (n = 4). (C) Macrophage morphology visualized by labelling with α‐actin (n = 4). (D) Images showing immunofluorescence of IL4, IL10, CD206, IFNγ and TNF‐α in macrophages (n = 4). The plotting scale is shown in the bottom right corner of each image. Values are the means ± SD values. *P < .05, **P < .01, compared with the control group

We then performed co‐culture experiments to explore the effect of Hoxa5 on co‐ordinating macrophages and adipocytes. The result showed that mRNA expression of TNF‐α and IL6 was decreased in the adipocytes which had been co‐cultured with the Hoxa5‐overexpressing macrophages (Figure 4A; *P* < .05). By activating PPARγ with the agonist, rosiglitazone in M1 macrophages, we found that overexpression of Hoxa5 up‐regulated mRNA expression of PPARγ, IL4 and CD206, and down‐regulated TNF‐α mRNA expression. In adipocytes, rosiglitazone enhanced the expression of PPARγ, IL4 and CD206 and reduced the expression of TNF‐α (Figure 4B; *P* < .05). In addition, we found that interference of PPARγ reversed the promotion effect of Hoxa5 on IL4 and CD163 (Figure S3B; *P* < .05). We then performed immunofluorescence staining of α *P* < 0 in protein to observe the effects of Hoxa5 on macrophage morphology and found that Hoxa5 reduced polarization of M1 macrophages, indicating an inhibitory effect (Figure 4C). Consistently, immunofluorescence staining results showed that overexpression of Hoxa5 increased the protein expression of IL4, IL10 and CD206 and decreased the protein expression of IFNγ (Figure 4D; *P* < .05). With regard to the in vivo experiment, we found that injection of adenovirus overexpressing Hoxa5 significantly decreased TNF‐α (*P* < .05) expression and increased the mRNA levels of IL4 (*P* < .05) and CD206 (*P* < .05) but had no significant effect on F4/80 expression in WAT from the LPS‐induced mouse model of inflammation (Figure S3A).

### 3.5 Hoxa5 increased polarized M2 macrophages by transcriptionally activating the PPARγ pathway

**Figure 5:**
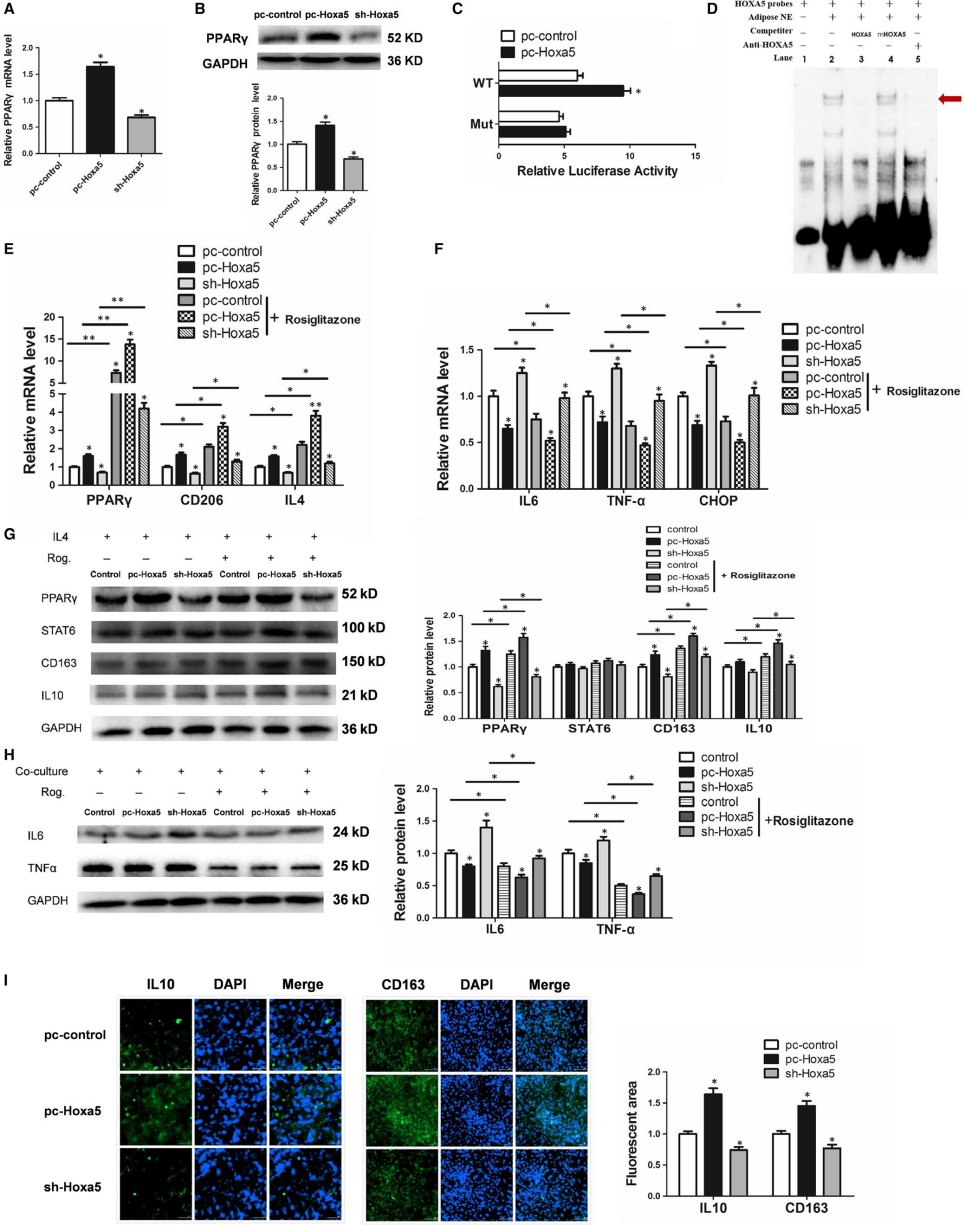
Hoxa5 increases M2‐polarized macrophages by transcriptional activating PPARγ pathway. (A‐B) Macrophages were transfected with pc‐Hoxa5 or sh‐Hoxa5. Relative mRNA or protein levels of PPARγ in macrophages (RAW264.7) (n = 4). (C) Mutational fragment of PPARγ promoter in a luciferase reporter vector in pc‐control or pc‐Hoxa5 groups (n = 4). (D) Electrophoretic mobility shift assay (EMSA) showing direct binding of Hoxa5 to the PPARγ promoter in vitro. The main complexes are marked with arrows. (E‐H) Macrophages were transfected with pc‐Hoxa5 or sh‐Hoxa5 with IL4 treatment and incubated with or without rosiglitazone. Adipocytes were co‐cultured with the macrophages from different groups. (E) Relative mRNA levels of PPARγ, CD206 and IL4 in macrophages (n = 4). (F) Relative mRNA levels of IL6, TNF‐α and CHOP in adipocytes (n = 4). (G) Relative protein levels of PPARγ, STAT6, CD163 and IL10 in macrophages (n = 4). (H) Relative protein levels of IL6 and TNF‐α in adipocytes (n = 4). (I) Images showing immunofluorescence of IL10 and CD163 in macrophages treatment with rosiglitazone (n = 4). Values are means ± SD values. *P < .05, **P < .01, compared with the control group

We then explored the regulatory role of Hoxa5 on macrophages, and the result showed that overexpression of Hoxa5 significantly increased PPARγ mRNA and protein expression in macrophages (Figure 5A, 5B; *P* < .05). Bioinformatics prediction results revealed a Hoxa5 binding site from −913 bp to −895 bp within the PPARγ promoter (Figure S4B). Using a luciferase reporter assay, we found that Hoxa5 may bind to this site and activate PPARγ expression (Figure 5C; *P* < .05). The EMSA experiment further confirmed that Hoxa5 binds to this motif of the PPARγ promoter (Figure 5D). The PPARγ agonist, rosiglitazone, promoted the expression of PPARγ, CD206 and IL4 mRNA in M2 macrophages, and Hoxa5 increased the expression of PPARγ, CD206 and IL4, regardless of the presence of rosiglitazone (Figure 5E; *P* < .05). The adipocytes co‐cultured with M2 macrophages overexpressing Hoxa5 treated with rosiglitazone exhibited down‐regulation of IL6, TNF‐α and CHOP mRNA expression, and PPARγ activation in the M2 macrophages inhibited the mRNA expression of IL6, TNF‐α and CHOP in the co‐cultured adipocytes (Figure 5F; *P* < .05). These results were consistent at the protein level, wherein Hoxa5 promoted the protein expression of PPARγ, CD163 and IL10 in M2 macrophages and inhibited the protein expression of IL6 and TNF‐α in the co‐cultured adipocytes (Figure 5G, 5H; *P* < .05). Notably, STAT6 signalling is not involved in Hoxa5‐dependent expression of PPARγ (Figure 5G). We next knocked down PPARγ in M2 macrophages and showed that knockdown of PPARγ reverses the effects of Hoxa5 on IL4 and CD163 expression (Figure S4B; *P* < .05). Immunofluorescence staining result showed that Hoxa5 increased the protein expression of IL10 and CD163 in M2 macrophages in the presence of rosiglitazone (Figure 5I; *P* < .05).

### 3.6 Hoxa5 relieved obesity‐induced chronic inflammation by promoting M1 to M2 *macrophages* polarization in adipose tissue of HFD mice

**Figure 6:**
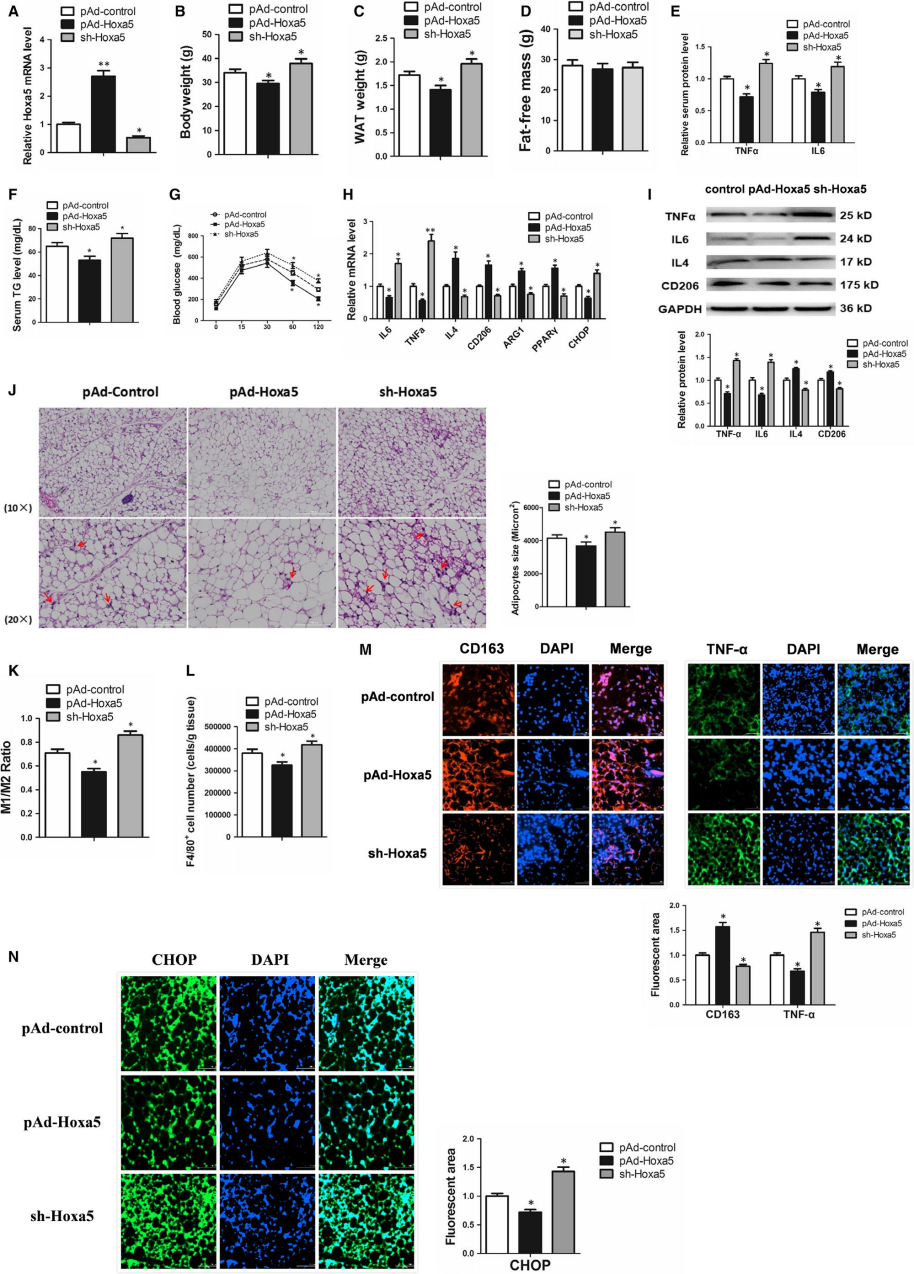
Hoxa5 relieves obesity‐induced chronic inflammation by promoting M1 to M2 macrophages polarization in adipose tissue of high‐fat diet (HFD) mice. HFD‐induced obesity mice were injected with pAd‐control, pAd‐Hoxa5 or sh‐Hoxa5. (A) Hoxa5 mRNA expression in mice iWAT (n = 4). (B) Bodyweight of mice in different groups (n = 6). (C) White fat mass of mice in different groups (n = 6). (D) Fat‐free mass of mice in different groups (n = 6). (E) Relative serum protein levels of TNF‐α and IL6 in mice (n = 4). (F) Relative serum triglycerides (TG) levels in mice (n = 6). (G) Glucose tolerance test (GTT) of the mice (n = 6). (H) Relative mRNA levels of IL6, TNF‐α, IL4, CD206, ARG1, PPARγ and CHOP in mouse iWAT (n = 6). (I) Relative protein levels of TNF‐α, IL6, IL4 and CD206 in mouse iWAT (n = 6). (J) Macrophage infiltration was monitored in frozen sections of iWAT samples by haematoxylin and eosin staining (n = 6). Red arrows show crown structures (CLS) of macrophages. (K‐F) SVF cells isolated from epididymal adipose tissue, abundances of all macrophages as well as those with the M1 and M2 phenotypes were analysed by flow cytometry. (K) The ratio of M1 (F4/80+CD11c+CD206−)/ M2 (F4/80+CD11c−CD206+) macrophages (n = 5). (L) The total number of F4/80^+^ cells that were isolated from epididymal adipose tissue (n = 5). (M) Images of iWAT from different groups that were immunofluorescent staining for CD163 and TNF‐α (n = 6). (N) Images of iWAT from different groups that were immunofluorescent staining for CHOP (n = 6). Values are the means ± SD values. *P < .05, **P < .01, compared with the control group

To determine the effect of Hoxa5 on the adipose tissue of obese mice, we injected adenovirus overexpressing Hoxa5 in high‐fat diet (HFD)–induced obese mice (Figure 6A; *P* < .01). The results showed that overexpression of Hoxa5 significantly reduced the bodyweight and WAT weight of obese mice (Figure 6B, 6; *P* < .05), while the average food intake and fat‐free mass have no significant difference between the two groups (Figure S5A, Figure 6D; *P* < .05). In addition, overexpression of Hoxa5 significantly decreased levels of inflammatory cytokines, TNF‐α and IL6, and triglycerides in serum (Figure 6E, 6F; *P* < .05). The GTT assay indicated that overexpression of Hoxa5 improved insulin sensitivity in mice (Figure 6G; *P* < .05). Analysis of mRNA expression in mouse inguinal WAT showed that overexpression of Hoxa5 down‐regulated the mRNA expression of IL6, TNF‐α and CHOP, while up‐regulated the expression of IL4, CD206, ARG1 and PPARγ, which is consistent with the in vitro data (Figure 6H; *P* < .05). Consistently, overexpression of Hoxa5 increased the protein expression of IL6 and TNF‐α, while decreased the expression of IL4 and CD206 (Figure 6I; *P* < .05). We further transplanted M2 macrophages into obese mice, which revealed that M2 macrophages increase the expression of IL4 and CD206 in adipose tissue, thereby reducing the expression of inflammatory cytokines, IL6 and TNF‐α, in adipose tissue (Figure S5B; *P* < .05). By H&E staining, we observed that overexpression of Hoxa5 reduced macrophage infiltration of mouse adipose tissue (Figure 6J). We further performed flow cytometric analyses of stromal vascular fractions (SVFs) of WAT in mice from different groups. The results showed that the ratio of M1 (F4/80+CD11c+CD206−)/ M2 (F4/80+CD11c−CD206+) macrophages was significantly lower in the mice of the pAd‐Hoxa5 group (Figure 6K; *P* < .05), and the total macrophages (F4/80+) were also decreased (Figure 6L; *P* < .05). Immunofluorescence staining showed an increase in CD163‐positive cells and a decrease in TNF‐α secretion, indicating the polarization of M2 macrophages in the pAd‐Hoxa5 group (Figure 6M; *P* < .05). And the result of immunofluorescence staining also showed that the expression of CHOP was inhibited by pAd‐Hoxa5 (Figure 6N; *P* < .05). In addition, we found there is no significant difference on the mRNA expression of WAT apoptosis marker genes (Bcl2, Bax, caspase 3) or browning marker genes (UCP1, PRDM16, Cidea) in different groups (Figure S5C, S5D), which suggested Hoxa5 reduced body fat mass by inhibiting reducing ER stress and inflammation instead of increasing apoptosis or WAT browning in this study. Taken together, these observations indicated that Hoxa5 reduces macrophage infiltration in WAT and increases polarization of M2 ATMs, thereby attenuating HFD‐induced chronic inflammation.

## 4 DISCUSSION

Numerous studies have reported that ATMs in healthy animals are selectively expressed in activated M2 macrophages. Furthermore, HFD induces obesity and increases the number of activated M1 macrophages, leading to the development of chronic inflammation in WAT and aggravated, systemic insulin resistance.^1^, ^36^, ^37^ Hoxa5 is an important transcription factor that is highly expressed in adipose tissue and plays a vital role in regulating adipocytes, including their differentiation and inflammation.^26^, ^27^ In this study, we observed that Hoxa5 relieved HFD‐induced ERS in WAT, and ERS aggravates M1 macrophage activation. This prompted us to explore the underlying relationships between Hoxa5, ERS and ATM polarization, thereby clarifying the role of Hoxa5 in the development of obesity and its regulatory mechanisms.

The ER acts as a central organelle in which transmembrane and secreted proteins are synthesized, folded and matured. Various genetic and environmental factors can lead to the accumulation of unfolded proteins in the lumen of the ER, causing ERS.^38^, ^39^ Eukaryotic cells have a system known as the unfolded protein response (UPR) to attenuate ERS, which is composed of three main sensors: PKR‐like ER kinase (PERK), inositol requires enzyme 1 (IRE1) and activating transcription factor 6 (ATF6).^40^ Furthermore, Hoxa5 promotes browning of white fat by inhibiting LPS‐induced inflammation and thus plays a positive role in fat metabolism.^26^ However, the role of Hoxa5 in obesity‐induced ERS is still unknown. Herein, RNA‐sequencing data revealed that overexpression of Hoxa5 in adipocytes resulted in differential expression of ER‐associated genes. PERK belongs to the eIF2α family, undergoes homodimerization and autophosphorylation, translocate to the Golgi apparatus, is activated^19^ and reduces cytotoxicity caused by erroneous protein folding by suppressing protein synthesis.^41^ Under ERS, PERK phosphorylates eIF2α,^42^ and then, p‐eIF2α induces expression of transcription factors, such as ATF4, to recover protein folding.^43^ Herein, we found that Hoxa5 reduced the expression of CHOP by inhibiting the PERK/eIF2α pathway and is accompanied by down‐regulation of inflammatory factor expression.

Furthermore, HFD‐induced up‐regulation of CHOP has been reported to be involved in the conversion of infiltrating ATMs to the Ml phenotype, and the expressions of IL‐4 and IL‐13 in adipocytes from WAT of CHOP‐/‐ mice are higher than in wild‐type mice.^22^ Here, considering the obvious inhibitory effect of Hoxa5 on CHOP, we also detected its effect on the polarization of macrophages in adipose tissue by performing co‐culture experiments of adipocytes and macrophages in vitro. We found that Hoxa5 increased polarization of M2 macrophages by inhibiting ERS in adipocytes.

It has been reported that Th2 cytokines (such as IL4 and IL10) activate M2 macrophages, and this process requires the participation of the transcription factor, PPARγ.^44^ Notably, STAT6 is involved in activating the PPARγ cascade in M2 macrophages in an IL4‐dependent manner.^45^ In the study, we determined that Hoxa5 transcriptionally activated PPARγ by EMSA and a luciferase assay, which resulted in increased polarization of M2 macrophages. In addition, we have excluded that Hoxa5 indirectly regulates PPARγ in a STAT6‐mediated manner. What is noteworthy is that the promotion of M2 polarization by Hoxa5 feedback relieved the inflammation and ERS in adipocytes, indicating a connection between macrophages and adipocytes. We also demonstrated the effect of M2 macrophages on inflammation and ER remission in primary adipose tissue from obese individuals by performing living transplantation. Moreover, injection of the Hoxa5 adenovirus vector into obese mice significantly relieved chronic inflammation and ERS in mouse adipose tissue, which was accompanied by polarization of M2 ATMs.

**Figure 7:**
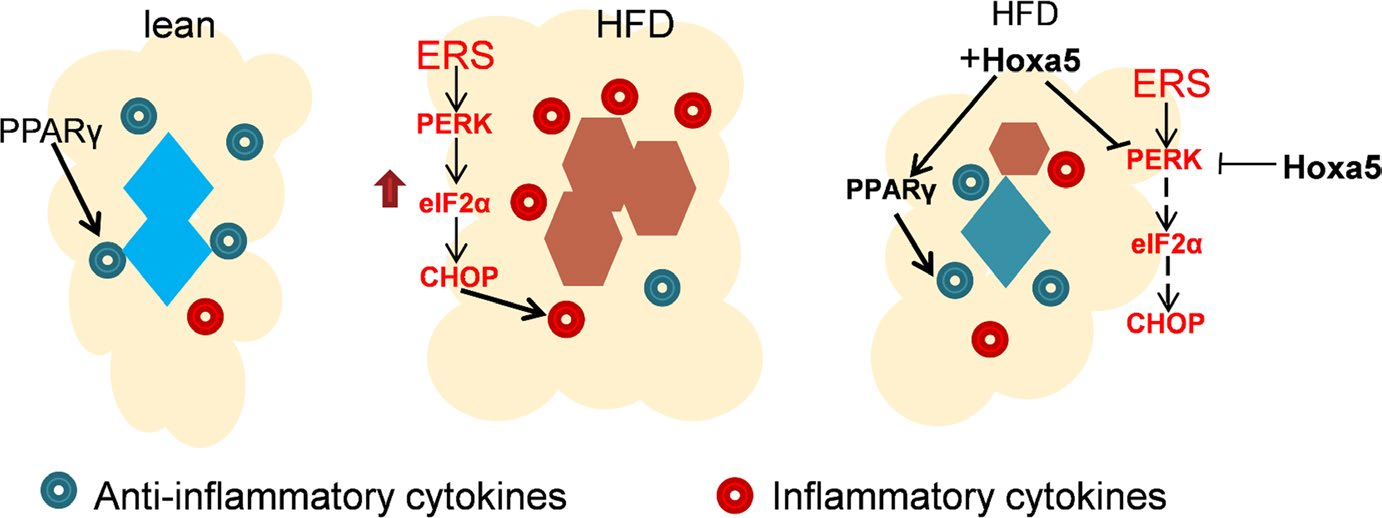
Hoxa5 alleviates obesity‐induced chronic inflammation by co‐ordinating adipocyte ER stress and polarization of M2 macrophages in mouse adipose tissue. Hoxa5 alleviates ER stress and inflammatory responses by inhibiting the eIF2α/PERK signalling pathway in adipocytes. Hoxa5 transcriptionally activates the PPARγ pathway to promote polarization of M2 macrophages, which in turn alleviates chronic inflammation of adipocytes

In conclusion, our findings revealed that Hoxa5 inhibits ERS by blocking the eIF2α/PERK signalling pathway in adipocytes and that Hoxa5 transcriptionally activated the PPARγ pathway in macrophages. This induces polarization of M2 macrophages, which in turn alleviates chronic inflammation in adipocytes (summarized in Figure 7). These findings suggest that Hoxa5 may be a potential therapeutic target for metabolic syndrome.

## CONFLICT OF INTEREST

The authors have no conflict of interest to declare.

## AUTHOR CONTRIBUTIONS

Weina Cao designed the study, collected the data, analysed the data, and wrote the manuscript; Tiantian Zhang and Ruonan Feng: collected the data, analysed the data; Hongtao Huang, Tianyu Xia and Chenlong Liu: analysed the data; and Chao Sun designed the study.

## ETHICAL APPROVAL

Mouse handling protocols were conducted following the guidelines and regulations approved by the Animal Ethics Committee of Northwest A&F University.

## Data Availability

The raw data have been deposited to NCBI Sequence Read Archive (SRA) under the accession code, SRP134917.
