# Supplementary figures and images for: Hoxa5 alleviates obesity‐induced chronic inflammation by reducing ER stress and promoting M2 macrophage polarization in mouse adipose tissue
Source: J Cell Mol Med. 2019 Aug 23;23(10):7029–42. doi: 10.1111/jcmm.14600 (PMC6787506; doi:10.1111/jcmm.14600)

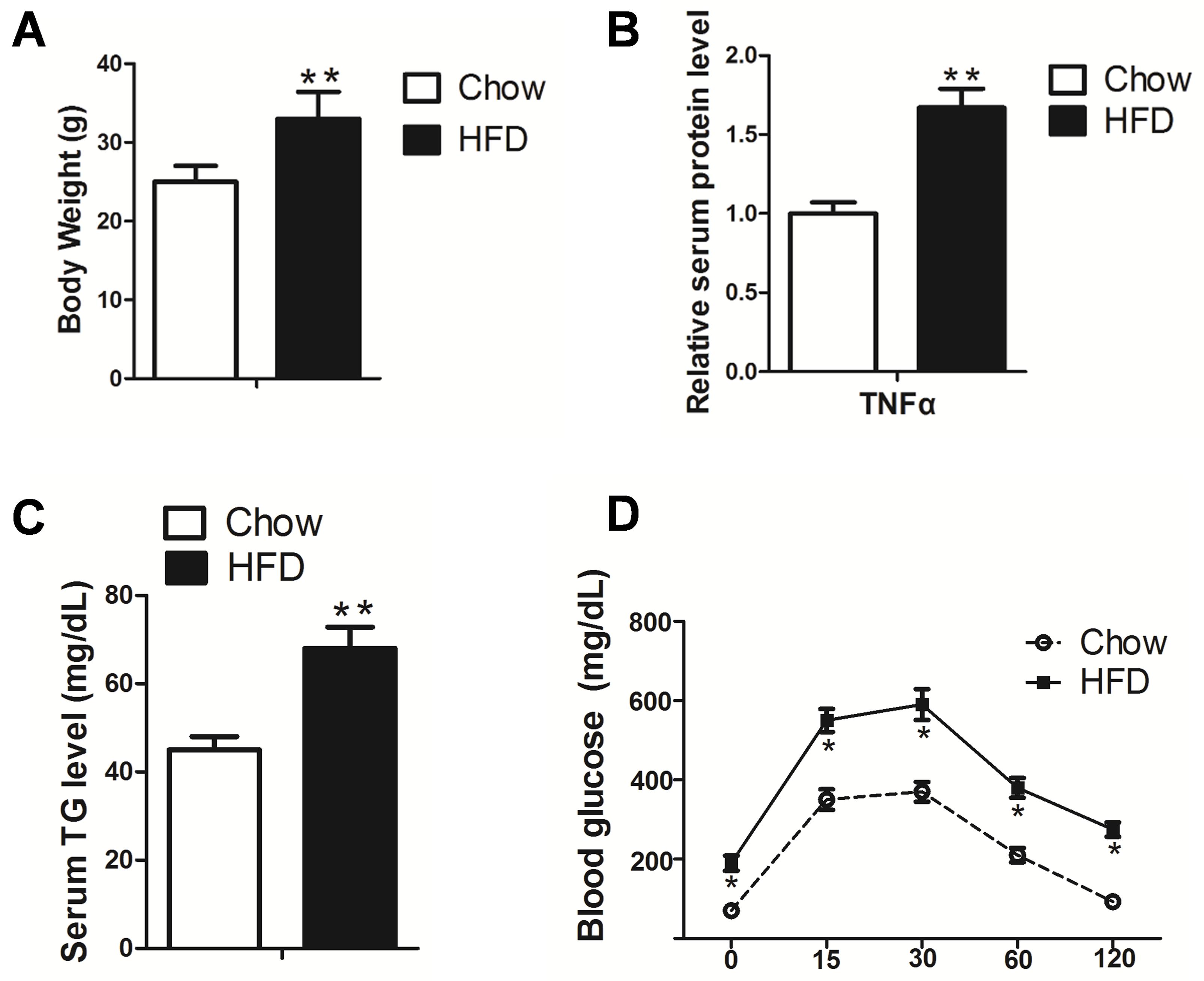

Supplement: Supplementary file 1 [file JCMM-23-7029-s001.jpg]

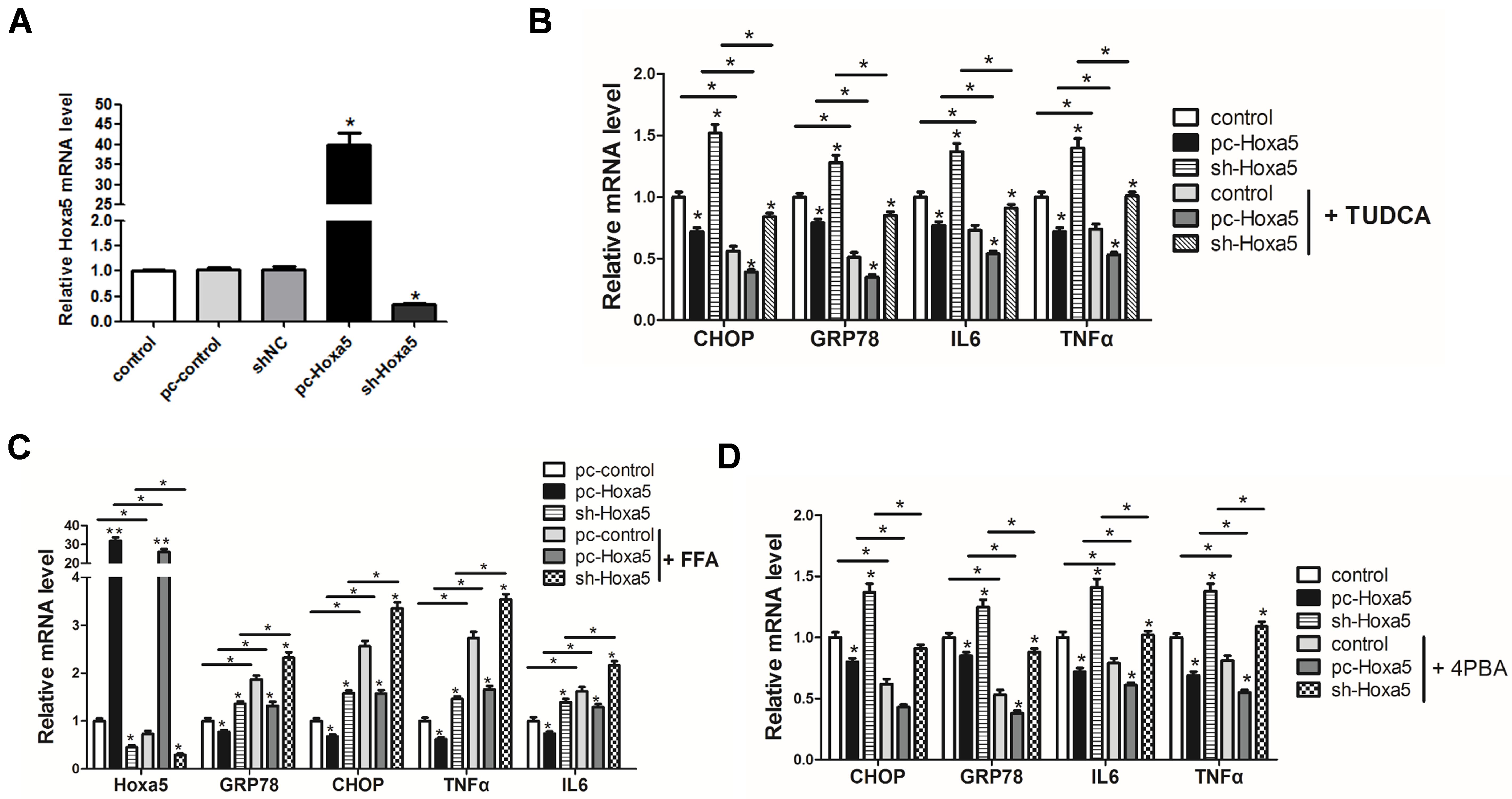

Supplement: Supplementary file 2 [file JCMM-23-7029-s002.jpg]

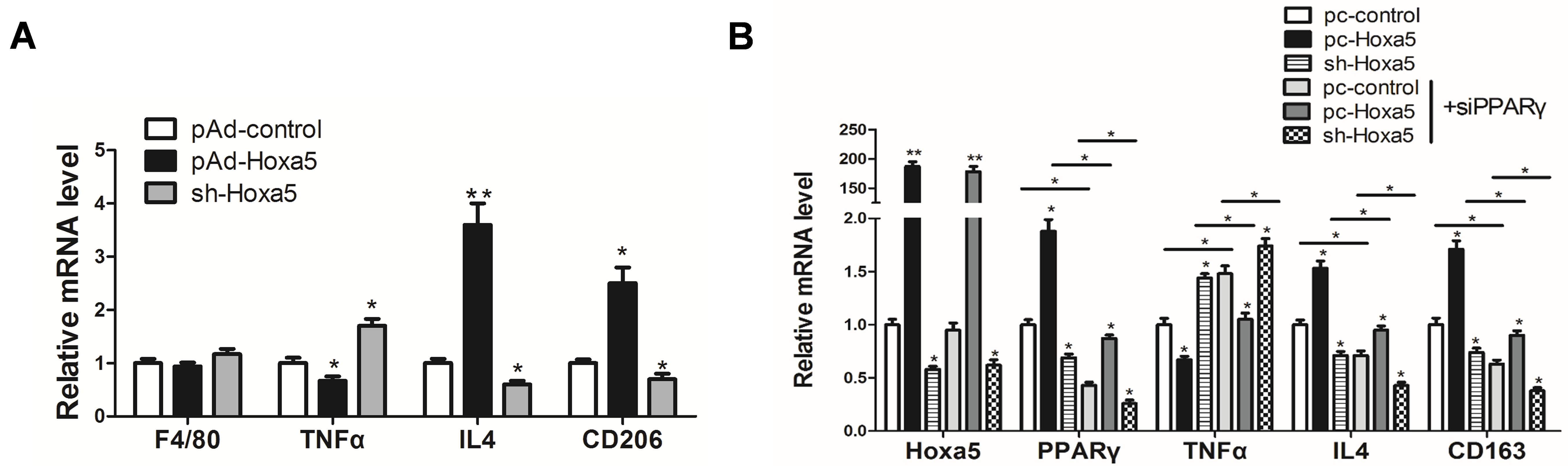

Supplement: Supplementary file 3 [file JCMM-23-7029-s003.jpg]

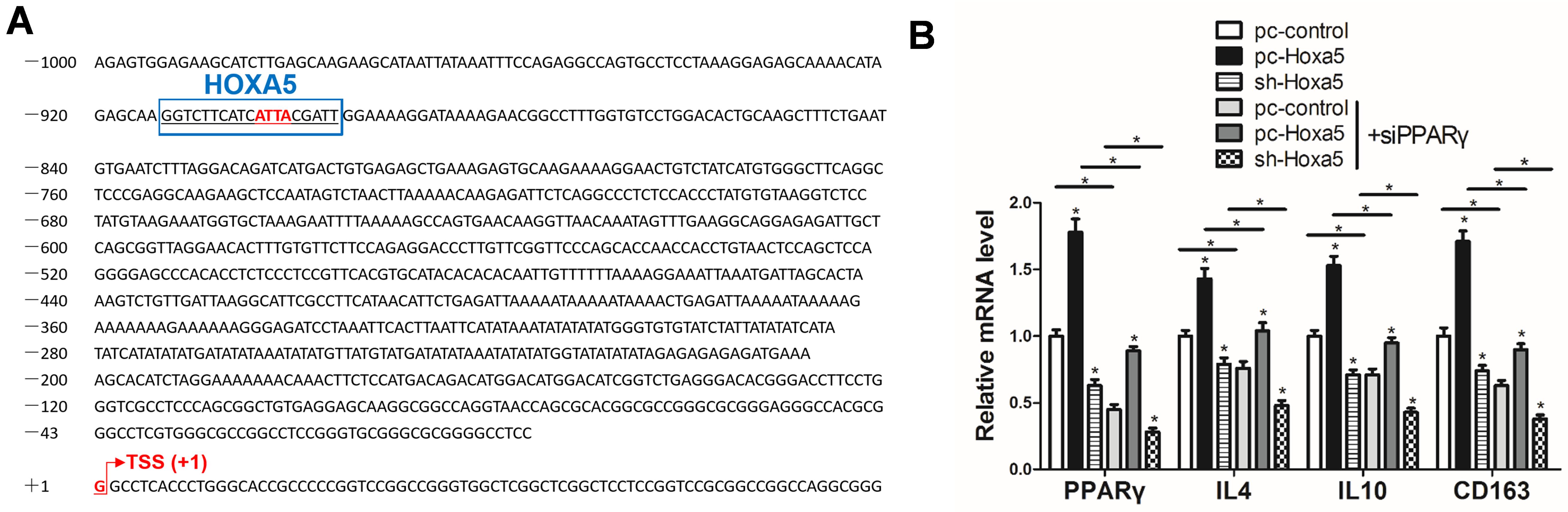

Supplement: Supplementary file 4 [file JCMM-23-7029-s004.jpg]

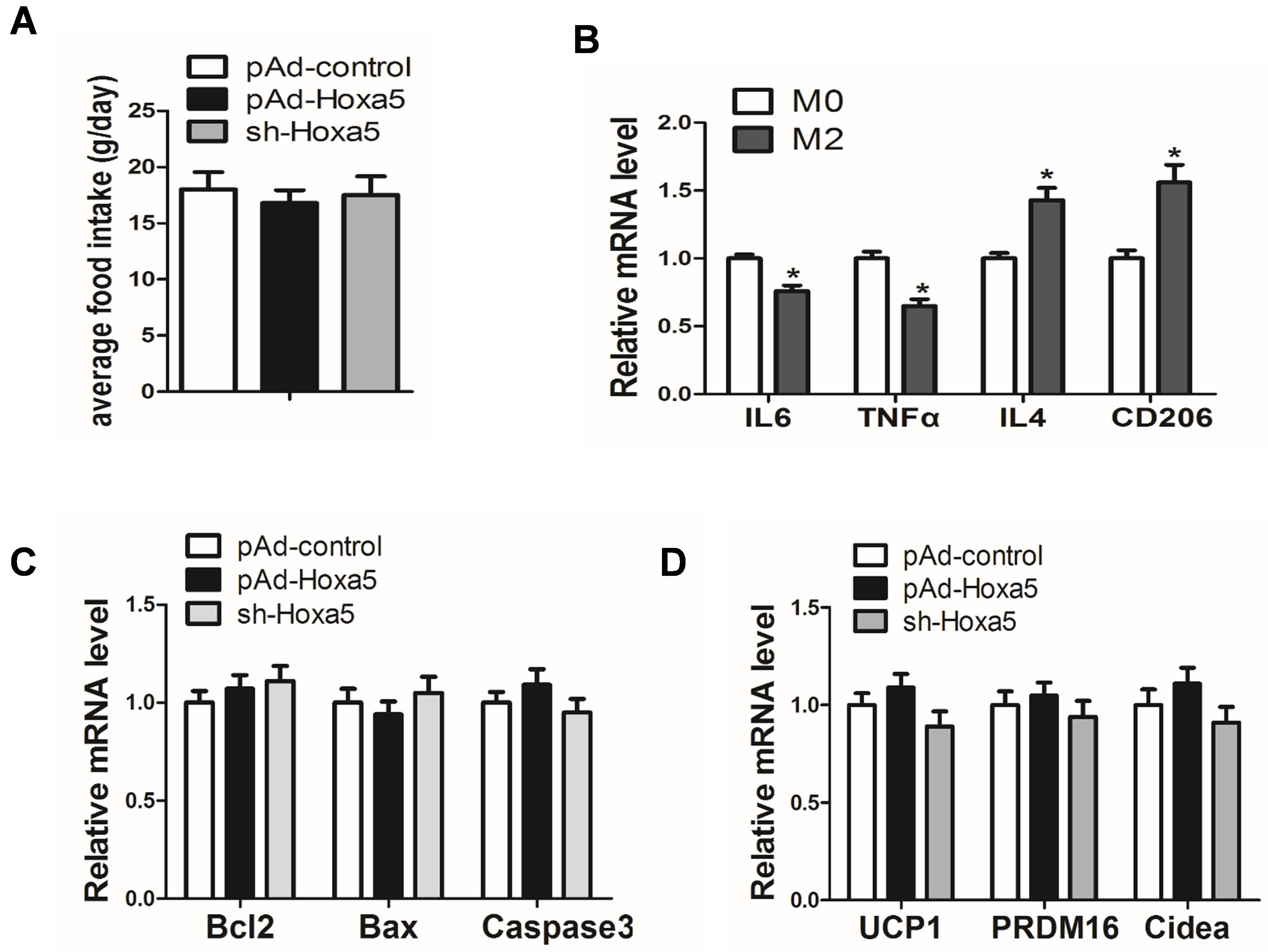

Supplement: Supplementary file 5 [file JCMM-23-7029-s005.jpg]
