# Supplementary material for: Hoxa5 alleviates obesity‐induced chronic inflammation by reducing ER stress and promoting M2 macrophage polarization in mouse adipose tissue
Source: J Cell Mol Med. 2019 Aug 23;23(10):7029–42. doi: 10.1111/jcmm.14600 (PMC6787506; doi:10.1111/jcmm.14600)
Supplement: Supplementary file 6 [file JCMM-23-7029-s006.doc]

**Supplementary Table:**

**Primers utilized in this study**

| **Reaction** | **Name** | **Primer Sequence (5′to 3′)** | **Tm (°C)** | **Product Length (bp)** | **Accession No** |
| --- | --- | --- | --- | --- | --- |
| **RT-PCR** | β-actin | F: AGCCATGTACCTAGCCATCC | 60.0 | 241 | NM_007393.5 |
|  |  | R: TTTGATGTCACGCACGATTT |  |  |  |
|  | Hoxa5 | F: CCCAGATCTACCCCTGGATG | 60.0 | 173 | NM_010453.5 |
|  |  | R: GGCATGAGCTATTTCGATCCT |  |  |  |
|  | CHOP | F: AACCTTCACTACTCTTGACCCTG | 60.0 | 226 | NM_007837.4 |
|  |  | R: CTCCTGCTCCTTCTCCTTCAT |  |  |  |
|  | GRP78 | F: ACCTATTCCTGCGTCGGTGT | 60.0 | 173 | XM_021194937.1 |
|  |  | R: GCGTCGAAGACCGTGTTCTCGG |  |  |  |
|  | MCP1 | F: CTGTGCTGACCCCAAGAAGGA | 60.0 | 182 | NM_011333.3 |
|  |  | R: TGGTTGTGGAAAAGGTAGTGG |  |  |  |
|  | TNFα | F: GTTCTCTTCAAGGGACAAGGCTG | 60.0 | 279 | NM_013693.3 |
|  |  | R: AATGACTCCAAAGTAGACCTG |  |  |  |
|  | IL6 | F: GCCTTCTTGGGACTGATGCTG | 60.0 | 171 | NM_031168.2 |
|  |  | R: CAACTCTTTTCTCATTTCCACG |  |  |  |
|  | PERK | F: TTGGGGACTTTGGACTGGTGAC | 60.0 | 218 | NM_010121.3 |
|  |  | R: CATCTGGGTGCTGAATGGGTAG |  |  |  |
|  | PPARγ | F: ACCACTCGCATTCCTTTGAC | 60.0 | 265 | NM_001127330.2 |
|  |  | R: CCACAGACTCGGCACTCAAT |  |  |  |
|  | F4/80 | F: CTTTGGCTATGGGCTTCCAGTC | 60.0 | 165 | NM_010130.4 |
|  |  | R: GCAAGGAGGACAGAGTTTATCGTG |  |  |  |
|  | CD163 | F: CCTCTGCTGTCACTAACGCTC | 60.0 | 142 | NM_001170395.1 |
|  |  | R: TCATTCATGCTCCAGCCGTTAC |  |  |  |
|  | CD206 | F: CCTCAACCCAAGGGCTCTTCTAA | 60.0 | 133 | NM_008625.2 |
|  |  | R: AGGTGGCCTCTTGAGGTATGTG |  |  |  |
|  | IL10 | F: TGGACAACATACTGCTAACCGAC | 60.0 | 111 | NM_010548.2 |
|  |  | R: CCTGGGGCATCACTTCTACC |  |  |  |
|  | XBP1 | F: ACATCTTCCCATGGACTCTG | 60.0 | 161 | NM_001271730.1 |
|  |  | R: TAGGTCCTTCTGGGTAGACC |  |  |  |
|  | ATF6 | F: AGACACTACCAGCCCTTATGCC | 60.0 | 264 | NM_001081304.1 |
|  |  | R: GTAGAACAGGTTTAGTCACGGA |  |  |  |
| **Site-mut and**  **EMSA** | Hoxa5 forward | GAGCAAGGTCTTCATC*ATTA*CGATTGGAAAAGGATA |  |  | −920/−885 |
|  | Hoxa5 reverse | TATCCTTTTCCAATCG*TAAT*GATGAAGACCTTGCTC |  |  |  |
|  | mHoxa5 forward | GAGCAAGGTCTTCATCCGATTGGAAAAGGATA |  |  |  |
|  | mHoxa5 reverse | TATCCTTTTCCAATCGGATGAAGACCTTGCTC |  |  |  |
